# Tuning Coupling Behavior of Stacked Heterostructures Based on MoS_2_, WS_2_, and WSe_2_

**DOI:** 10.1038/srep44712

**Published:** 2017-03-17

**Authors:** Fang Wang, Junyong Wang, Shuang Guo, Jinzhong Zhang, Zhigao Hu, Junhao Chu

**Affiliations:** 1 Technical Center for Multifunctional Magneto-Optical Spectroscopy (ECNU), Shanghai Department of Electronic Engineering, East China Normal University, Shanghai 200241, China

## Abstract

The interlayer interaction of vertically stacked heterojunctions is very sensitive to the interlayer spacing, which will affect the coupling between the monolayers and allow band structure modulation. Here, with the aid of density functional theory (DFT) calculations, an interesting phenomenon is found that MoS_2_-WS_2_, MoS_2_-WSe_2_, and WS_2_-WSe_2_ heterostructures turn into direct-gap semiconductors from indirect-gap semiconductors with increasing the interlayer space. Moreover, the electronic structure changing process with interlayer spacing of MoS_2_-WS_2_, MoS_2_-WSe_2_, and WS_2_-WSe_2_ is different from each other. With the help of variable-temperature spectral experiment, different electronic transition properties of MoS_2_-WS_2_, MoS_2_-WSe_2_, and WS_2_-WSe_2_ have been demonstrated. The transition transformation from indirect to direct can be only observed in the MoS_2_-WS_2_ heterostructure, as the valence band maximum (VBM) at the Γ point in the MoS_2_-WSe_2_ and WS_2_-WSe_2_ heterostructure is less sensitive to the interlayer spacing than those from the MoS_2_-WS_2_ heterostructure. The present work highlights the significance of the temperature tuning in interlayer coupling and advance the research of MoS_2_-WS_2_, MoS_2_-WSe_2_, and WS_2_-WSe_2_ based device applications.

Semiconductor heterojunctions have played a significant role in the rich collection of unimagined electronic structures and optical properties^1^. The emergence of atom-thin transition-metal dichalcogenides (TMDs) as a new class of two dimensional semiconducting materials, which are almost as thin, transparent, and flexible as graphene with a wealth of new physical phenomena^2^,^3^,^4^,^5^, creates exciting new opportunities to push semiconductor heterostructures toward a new frontier^6^,^7^. Vertically stacked van der Waals TMDs heterostructures have been recognized as a powerful platform to create atomically thin heterostructures^8^,^9^,^10^. These heterojunctions have an optically active band gap with bound electrons and holes localized in individual monolayers^11^,^12^, and their energy and luminescence intensity are highly tunable with the applied vertical gate voltage, laser intensity, and annealing time^12^,^13^. A more desirable way to control the interlayer coupling properties is adjusting the interlayer spacing, which is closely related with the temperature^14^,^15^. Temperature induces lattice parameter changes and modulates the band structure, altering the optical properties of the heterojunctions. Further more, it is important to investigate the temperature induced phenomena in order to distinguish the phenomena influenced by other factors, such as the surface quality, the strain applied on the heterostructures, and even the number of atomic layers, etc. However, such an interesting and important research of variable-temperature tuning for TMDs heterostructures has not been reported up to now.

In this article, high-quality MoS_2_-WS_2_, MoS_2_-WSe_2_, and WS_2_-WSe_2_ heterostructures have been fabricated by the polystyrene film transferred technique. They are systematically studied by tuning interlayer coupling with temperature for the first time. Furthermore, it is discovered that the temperature is an extremely sensitive factor to the interlayer coupling in comparison with the annealing tuning. Based on the density functional theory (DFT) calculations, an interesting phenomenon is found that MoS_2_-WS_2_, MoS_2_-WSe_2_, and WS_2_-WSe_2_ heterostructures turn into direct-gap semiconductors from indirect-gap semiconductors with increasing the interlayer space. However, in the MoS_2_-WS_2_, MoS_2_-WSe_2_, and WS_2_-WSe_2_ heterostructures, the change of the bandgap properties with interlayer spacing differs from each other. The results has been observed in the variable-temperature experiment. Our results not only compare the MoS_2_-WS_2_, MoS_2_-WSe_2_, and WS_2_-WSe_2_ heterostructures tuning character, but also open up a new direction for 2D applications where external modulation of bandgap and optical properties is desired.

## Results and discussion

### Raman scattering

**Figure 1:**
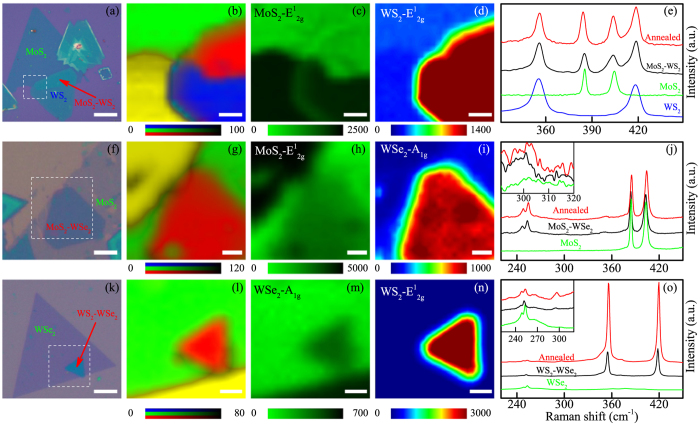
TMDs heterostructures prepared by the PS film transfer technique. (**a,f,k**) Optical microscope image of MoS_2_-WS_2_, MoS_2_-WSe_2_, and WS_2_-WSe_2_ heterostructures. (**b,g,l**) Raman mapping in the confocal measurements for the heterostructures in the dashed frame region in panels (a), (f), and (k). The yellow parts correspond to the substrates, the green, blue parts refer to the single TMDs regions, and red parts refer to heterogeneous regions. (**c**,**h**,**m**) Single color mapping of Raman intensity at the 385 cm^−1^ (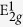 mode in MoS_2_) and 356 cm^−1^ (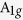 mode in WSe_2_). (**d**,**i**,**n**) Pseudo-color mapping of Raman intensity at 356 cm (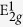 mode in WS_2_) and 254 cm^−1^ (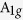 mode in WSe_2_). (**e**,**j**,**o**) The Raman spectra of the MoS_2_-WS_2_, MoS_2_-WSe_2_, and WS_2_-WSe_2_ heterostructures corresponding to the different region in panels (b), (g), and (l). Scale bars of panels (a), (f), and (k) are 20 *μ*m, 4 *μ*m, and 6 *μ*m, respectively. Scale bar of panels (b-d) are 4 *μ*m; that of panels (**g–i**) and (**l–n**) are 2 *μ*m. The colour scale bars in the bottom of (**b**–**d**), (**g**–**i**), and (**l**–**n**) correspond to the intensity of the Raman spectra.

The vertically stacked heterostructures have been investigated by Raman scattering and PL spectroscopy. The optical images and Raman spectra are shown in Fig. 1. It indicates that the MoS_2_-WS_2_, MoS_2_-WSe_2_, and WS_2_-WSe_2_ heterostructures have been stacked successfully. The monolayer and heterostructure regions can be readily distinguished under the optical microscope and Raman mapping.

The 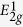 and A_1*g*_ Raman modes are located at 385.1 cm^−1^ and 404.4 cm^−1^ for monolayer MoS_2_ [green line in Fig. 1(e)], whereas those for monolayer WS_2_ are 356.1 cm^−1^ and 417.4 cm^−1^ [blue line in Fig. 1(e)]^16^,^17^,^18^. The Raman spectra recorded on the MoS_2_-WS_2_ heterostructure [black line in Fig. 1(e)] seem to be a simple superposition of the monolayer MoS_2_ and WS_2_. Even after the annealing at 100 °C in argon gas for 6 hours, except for increasing the intensity [red line in Fig. 1(e)]^13^. The frequency difference between the 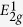 and A_1*g*_ Raman modes is 18.6 cm^−1^ for monolayer MoS_2_ in MoS_2_-WSe_2_ [green line in Fig. 1(j)], and 4.7 cm^−1^ for monolayer WSe_2_ in WS_2_-WSe_2_ [green line in Fig. 1(o)], respectively^19^. While in the WSe_2_-based heterostructures, an additional weak Raman peak pointed to interlayer coupling near 300 cm^−1^ can be observed [insert figures in Fig. 1(j) and (o)], which disappears in the monolayer WSe_2_. It corresponds to the B_2*g*_ resonance mode of WSe_2_^20^. In general, the B_2*g*_ signature mode is only active in the bilayer or few-layer WSe_2_, which could reflect the presence of the additional interlayer interaction in the present TMDs heterostructures^20^. It demonstrates that the B_2*g*_ mode is more sensitive to interlayer interaction than the out-of-plane modes A_1*g*_^19^,^20^,^21^.

### Band structures for heterostructures

**Figure 2:**
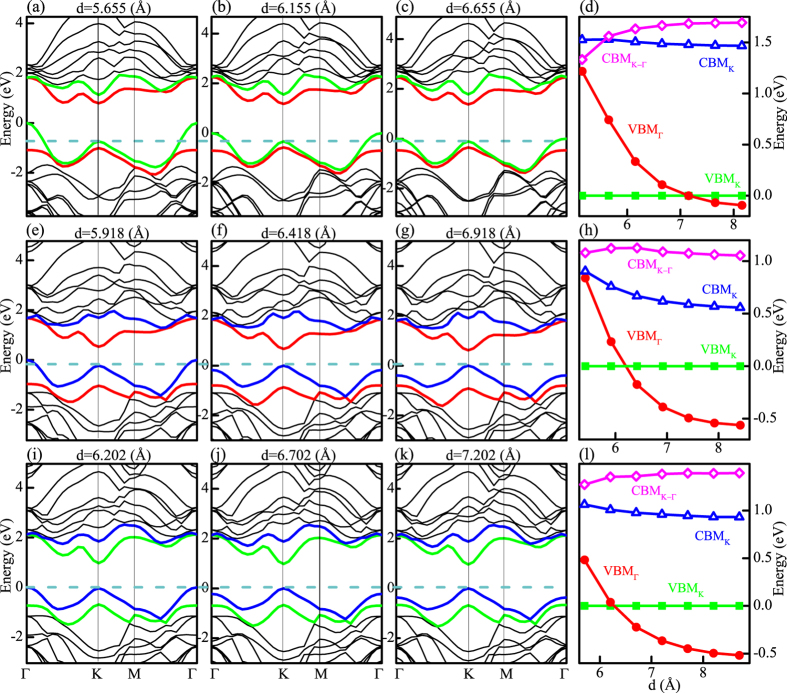
The band structures for MoS_2_-WS_2_, MoS_2_-WSe_2_, and WS_2_-WSe_2_ heterostructures. (**a–c**) Band structures for MoS_2_-WS_2_ when the layer spacing is (**a**) 0.5 Å smaller than the equilibrium distances, (**b**) equal to the equilibrium distances, (**c**) and 0.5 Å more than the equilibrium distances. (**e–g**) Band structures for MoS_2_-WSe_2_ when the layer spacing is (**e**) 0.5 Å smaller than the equilibrium distances, (**f**) equal to the equilibrium distances, and (**g**) 0.5 Å more than the equilibrium distances. (**i–k**) Band structures for WS_2_–WSe_2_ when the layer spacing is (**i**) 0.5 Å smaller than the equilibrium distances, (**j**) equal to the equilibrium distances, and (**k**) 0.5 Å more than the equilibrium distances. (**d,h,l**) Variation of the VBM and CBM with respect to the the layer spacing for (**d**) MoS_2_-WS_2_, (**h**) MoS_2_-WSe_2_, and (**l**) WS_2_-WSe_2_. VBM_*K*_ and CBM_*K*_ refer to the local highest valence and lowest conduction states at K point, VBM_Γ_ to the Γ point, and CBM_*K*−Γ_ to the path K-Γ.

The conduction band minimum (CBM) and valence band maximum (VBM) at the K point are primarily composed of the Mo or W 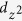 states, while the CBM at K-Γ and the VBM at Γ are dominated by the Mo or W 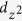 and and S *p* states. The Mo 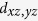 states are far away from the Fermi energy^13^. Because of different orbital character, the highest valence and lowest conduction states respond very differently to the interlayer spacing and coupling. For a detailed research for the interlayer spacing and coupling, the DFT calculations for MoS_2_-WS_2_, MoS_2_-WSe_2_, and WS_2_-WSe_2_ heterostructures have been carried out, which are shown in Fig. 2.

The equilibrium interlayer separation distances with geometry optimization for MoS_2_-WS_2_, MoS_2_-WSe_2_, and WS_2_-WSe_2_ heterostructures are 6.155 Å, 6.418 Å, and 6.702 Å, respectively. The corresponding band structures near the equilibrium layer spacing are shown in Fig. 2(a–c) (MoS_2_-WS_2_), Fig. 2(e–g) (MoS_2_-WSe_2_), and Fig. 2(i–k) (WS_2_-WSe_2_). Interestingly, at the equilibrium layer spacing of 6.155 Å in the MoS_2_-WS_2_ heterostructure, the VBM is at the Γ point because of the interaction between layers [Fig. 2(b)]. This results in an indirect transition from the VBM at the Γ point to the CBM at the K points. It is different from the direct transition situated at K point of the monolayer TMDs. The VBM at the Γ point becomes even higher to the K point when the interlayer separation distances is reduced to 5.655 Å in Fig. 2(a). The results are more obvious in Fig. 2(d), which shows the variations of the highest valence and lowest conduction states as functions of the layer spacing in MoS_2_-WS_2_. It can be seen that the reduction in the interlayer spacing modifies the VBM and CVM values at high symmetry points effectively. As the VBM at the Γ point involving *p-d* orbital coupling, it changes dramatically. As a result, the indirect bandgap increases obviously for shorter layer spacing. With decreasing the layer spacing from 7.155 Å to 5.155 Å, not only the VBM at Γ point becomes higher with respect to the K point, but also the CBM at a midpoint between K and Γ points becomes lower than that at the K point. However, the direct excitonic transition energy at the K point shows small changes.

The band structures for MoS_2_-WSe_2_ [Fig. 2(e–h)] and WS_2_-WSe_2_ [Fig. 2(i–l)] are quite different from for MoS_2_-WS_2_ heterostructure. The MoS_2_-WSe_2_ and WS_2_-WSe_2_ show direct gaps at the K point around 1 eV at the equilibrium layer spacing^22^, and the VBM at the Γ point changes less obviously than the MoS_2_-WS_2_ heterostructure. Especially for the WS_2_-WSe_2_ heterojunction, even though the interlayer separation distances is reduced 0.5 Å from the equilibrium layer spacing, the indirect transition is not as obvious as the other two heterostructures. Moreover, by varying the interlayer distance from 7.702 to 5.702 Å, the VBM at the Γ point for WS_2_-WSe_2_ changes only 1 eV, which is less than those of about 1.3 eV in the MoS_2_-WS_2_ heterostructure. This phenomenon indicates that the interlayer coupling tuning with interlayer separation distances for WS_2_-WSe_2_ heterostructure is relatively difficult, which is in accordance with the presenting experimental results.

### Variable-temperature tuning process

**Figure 3:**
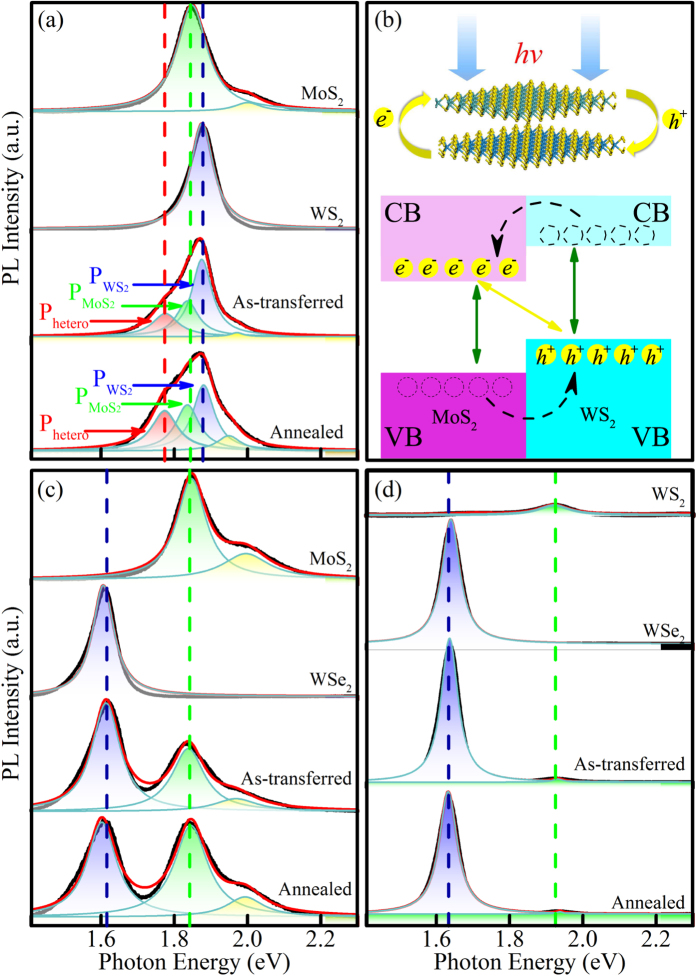
(**a**) Multiple-peak fitting to measured PL spectra of MoS_2_, WS_2_, as-transferred, and annealed MoS_2_-WS_2_ heterostructures. (**b**) Schematic of the band alignment at the K-point for the MoS_2_-WS_2_ heterostructure. (**c**) Multiple-peak fitting to measured PL spectra of MoS_2_, WSe_2_, as-transferred, and annealed MoS_2_-WSe_2_ heterostructures. (**d**) Multiple-peak fitting to measured PL spectra of WS_2_, WSe_2_, as-transferred, and annealed WS_2_-WSe_2_ heterostructures. Lorentz Model was used in the fitting to the PL spectra.

From tight-binding theory or quantum tunneling model, the interlayer interaction is expected to be exponentially sensitive to the interlayer distance. In order to further confirm the theoretical calculation and investigate the interlayer coupling with different interlayer distance, variable-temperature tuning process were performed in MoS_2_-WS_2_, MoS_2_-WSe_2_, and WS_2_-WSe_2_ heterostructures. It has been known that the physical properties of the heterostructures could be affected by the twist angle between the two layers as observed in graphene and graphitic materials^23^,^24^. To avoid the interference of the twist angle, the whole variable-temperature experiment was carried on the same point of the heterostructure. For the purpose of obtaining information on the band properties of the heterojunctions, optical properties are characterized with PL spectroscopies in Fig. 3.

All MoS_2_-WS_2_, MoS_2_-WSe_2_, and WS_2_-WSe_2_ heterostructures form a type II band alignment^22^,^25^. In type II heterojunctions, the CBM and VBM reside in two separate materials. Moreover, the type II band alignment and built-in potential in the heterojunctions can facilitate the photoexcited electron-hole separation and lead to an enhanced photoswitching performance compared to that in MoS_2_ WS_2_ and WSe_2_^26^. For example, in MoS_2_-WS_2_ heterostructure, due to the type-II band alignment, photoexcited electrons and holes will relax (dashed lines in Fig. 3(b)) to the conduction band edge of MoS_2_ and the valence band edge of WS_2_, respectively. The coulomb attraction between electrons in MoS_2_ and holes in WS_2_ gives rise to an interlayer exciton 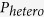. It is analogous to spatially indirect excitons in coupled quantum wells^27^. Therefore, a new peak at 1.77 eV is observed 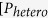 in Fig. 3(a)], lying interestingly at a lower energy than the peak for the two constituent single layers. It is noteworthy that the relative intensity for 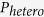 and MoS_2_, WS_2_ changed after the annealing progress. The relative intensity of 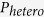 to 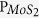 is expressed as 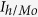, 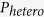 to 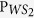 is 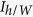. After the annealing progress, 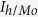 increases from 0.65 to 0.87, 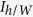 increases from 0.3 to 0.61. Such a pronounced luminescence effect in the heterostructure suggests that most electrons excited in WS_2_ transfer to the lower states in MoS_2_, instead of forming excitons in WS_2_ and recombine radiatively. The annealing progress promote the electrons transfer in the heterostructure, as the annealing is able to drive out trapped residual molecules^28^. However, in equilibrium condition of the MoS_2_-WSe_2_ and WS_2_-WSe_2_ heterostructures, the direct gaps at the K point are around 1 eV [Fig. 2(f) and (j)].which are different from the indirect transition of MoS_2_-WS_2_^22^. Under these conditions, the PL spectra from 1.4 eV to 2.3 eV for MoS_2_-WSe_2_ and WS_2_-WSe_2_ heterostructures in Fig. 3(c) and (d) appear to comprise the addition of the constituent layers.

**Figure 4:**
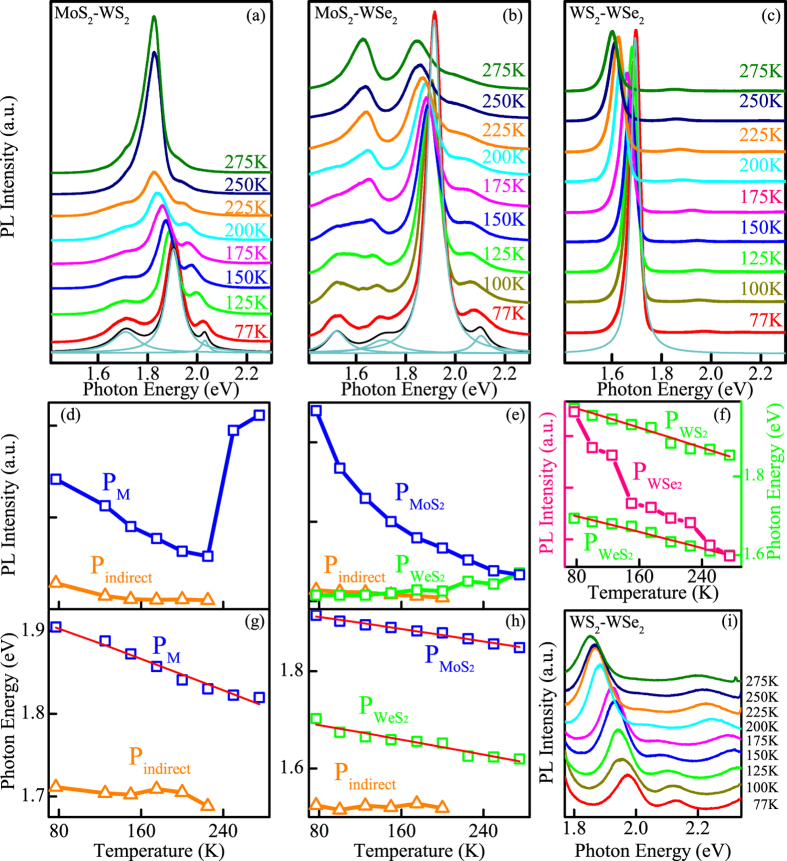
Temperature dependent PL of heterostructures (**a**) MoS_2_-WS_2_. (**b**) MoS_2_-WSe, (**c**) and WS_2_-WSe_2_. In the bottom of the spectra (**a**), (**b**), and (**c**), the Lorentzian fit functions to the spectra are shown. (**d**) Fitted PL intensity and (**g**) peak position of the direct transition integrated intensity P_*M*_ and indirect transition P_*indiret*_ versus temperature in MoS_2_-WS_2_ heterostructure. (**e**) Fitted PL intensity and (**h**) peak position of the 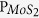, 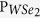, and P_*indiret*_ in MoS_2_-WSe_2_ heterostructure. (**f**) Fitted PL Peak position and intensity of the 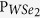 in WS_2_-WSe_2_ heterostructure. (**i**) Enhanced temperature dependent PL spectra of WS_2_-WSe_2_ heterostructure between 1.77 eV and 2.34 eV.

In the variable-temperature tuning process for the MoS_2_-WS_2_, the excitonic transitions of MoS_2_ (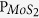), WS_2_ (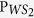), and interlayer exciton (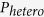) are fitted with the integrated intensity peak 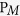, with the purpose of describing the intensity change with temperature. Figure 4(a) shows the luminescence evolutions of the MoS_2_-WS_2_ heterostructure as a function of temperature from 77 to 275 K with a stability of about 0.1 K. When the excitonic transitions of MoS_2_ (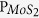), WS_2_ (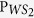), and interlayer exciton (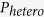) are fitted with the integrated intensity peak 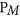, the PL spectra can be deconvoluted into three major peaks when the temperature is below 225 K. The resonance at about 2 eV corresponds to the B exciton, which attributes to the top of valence-band splitting due to the strong spin-orbital interaction in MoS_2_^29^. In addition to the above excitons, another transition at a lower energy can be observed at about 1.7 eV, which seems different from the previous work^30^,^31^. The transition becomes negligible in the temperature above 250 K [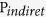 in Fig. 4(a),(d) and (g)]. This characteristic is profoundly different from the behaviors of other double layer transition metal sulfides. Nanostructures obtained from indirect bandgap transition metal sulfides emit an indirect transition excitons upon photoexcitation, but the luminescence is present in the room temperature as well (Figure S1)^32^. In order to identify the peak 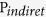, another significant change should be noticed. When the temperature increases above 250 K, the intensity of the peak 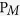 increases dramaticly by about three times. The integral PL intensity generally decreases with increasing the temperature. The phenomena can be observed in temperature dependent PL measurement of monolayer MoS_2_, as shown in Figure S2. This quenching effect is due to that the nonradiative electron-hole recombination rate increases exponentially with increasing the temperature^33^,^34^. The nonradiative channels, such as trapping by surface/defect/ionized impurity states, become thermally activated with increasing the temperature and the nonradiative lifetime *τ*_NR_ is expressed as ref. 35


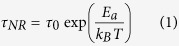


where *τ*_0_ is the pre-exponential factor, *E*_*a*_ is the activation energy in the thermal quenching process and *k*_*B*_ is the Boltzmann constant. When the temperature increases, the decreasing *τ*_*NR*_ results in a decrease in the luminescence intensity, which can be expressed as ref. 36


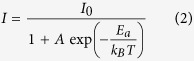


where *I*_0_ is the intensity at 0 K, *A* is a parameter related to radiative lifetime (*τ*_*R*_) as *A* = *τ*_*R*_/*τ*_0_, and *τ*_*R*_ is the radiative lifetime. However, the peak P_*M*_ reveals the monotonic decrease of PL intensity with increasing the temperature only when the temperature is below 250 K, and the PL intensity is even stronger when the temperature is above 250 K in spite of the increasing temperature.

In order to explain the extraordinary change of the P_*M*_ intensity, the luminescence quantum efficiency is introduced. As the luminescence physics mechanism for transition metal sulfide is different from traditional silicon nanocrystals. In silicon nanocrystals, the photoluminescence originates from quantum confined electronic states with increased emission energy at decreased nanoparticle size. The radiative transition rate is quite low. In contrast, luminescence for transition metal sulfide arises from electronic transitions, which shows a much higher radiative recombination rate. Therefore, the extraordinary change of the P_*M*_ intensity is explained by luminescence quantum efficiency, which is approximated by ref. 37


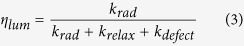


where *k*_*rad*_ is the rates of radiative recombination, *k*_*relax*_ is electron relaxation within the conduction and valence bands, and *k*_*defect*_ is defect trapping, respectively. The *k*_*red*_ is not likely to change appreciably with the change of the interlayer spacing in heterostructure, because the direct excitonic transitions at Γ point do not show significant change. The *k*_*defect*_ is stable relatively. Therefore, when the temperature increases above 250 K, the significant enhanced luminescence of P_*M*_ has to be attributed to the dramatically reduced electronic relaxation *k*_*relax*_. The decrease of interband relaxation rate strongly suggests a substantial change in the MoS_2_-WS_2_ heterostructure electronic structure when the temperature increases from 225 K to 250 K. It is noteworthy that the value of the phonon-assisted *k*_*relax*_ is very large for the indirect bandgap semiconductor. Because when the indirect bandgap disappears, the decay rate via phonons decreases enormously^32^,^37^. As a consequence, when the temperature increases from 225 K to 250 K, the indirect bandgap disappears. The substantial change is in accordance with the transition transformation analysed in the DFT calculations. The indirect transition from the VBM at Γ point to the CBM at K point disappeared with increasing the interlayer spacing. Based on the above analysis, the transition P_*indiret*_ disappeared above 250 K is attributed to indirect band gap emission.

It is noteworthy that, the MoS_2_-WSe_2_ prefers direct transition at the equilibrium layer spacing, and the VBM at the Γ point for the MoS_2_-WSe_2_ band structure changes less obviously than the MoS_2_-WS_2_ [Fig. 2(e–h)]. In the luminescence evolutions of MoS_2_-WSe_2_ heterostructure [Fig. 4(b)], another indirect transition peak P_*indiret*_ emerges at the temperature below 225 K [Fig. 4(e)]. However, the luminescence trends for the 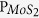 and 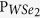 are different from MoS_2_-WS_2_. Among the heterostructures studied, the atomic differences in MoS_2_-WSe_2_ are the most obvious. Both the transition metals and the sulfur family elements are different. As a consequence, the WSe_2_ attributed part in the band structure is significantly influenced in the heterostructure [blue line in Fig. 2(f)]. An indirect transition from the VBM of WSe_2_ to the WSe_2_ induced conduction band between Γ and K points is caused. Moreover, the P_*indiret*_ is quenching with increasing the temperature [Fig. 4b and e)], as the influence in the band structure of the WSe_2_ attributed part declines when the interlayer spacing increases. Along with the quenching of the indirect transition, the direct transition excitons increase, and the 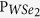 increases gradually. However, the VBM at the Γ point in the MoS_2_-WSe_2_ heterostructure is less sensitive to the interlayer spacing than the MoS_2_-WS_2_ heterostructure[Fig. 2]. Therefore, the dramatically increasing intensity of the PL has not been observed here.

Not only for MoS_2_-WSe_2_, but the VBM at the Γ point for WS_2_-WSe_2_ heterostructure is insensitive to the interlayer spacing [Fig. 2(l)]. Moreover, the indirect transition for WSe_2_ in the WS_2_-WSe_2_ is less obvious than that in the MoS_2_-WSe_2_ heterostructure. As a consequence, the 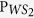 and 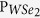 present an redshift for conventional semiconductor. In addition, it is important to note that the B exciton peak of WS_2_ and WSe_2_ can be observed in the enhanced PL spectra of WS_2_-WSe_2_ heterostructure in Fig. 4(i). The peaks near 1.98 eV and above 2.3 eV at 77 K are attributed to be the A and B excition of WS_2_^17^,^32^. The peak near 2.13 eV at 77 K corresponds to the B excition of WSe_2_. It is 430 meV higher than the A excition, which is in agreement with the previous reports^32^. The B exciton peak for WS_2_ and WSe_2_ with such an intensity is rarely observed in the previous PL measurement. What is more, it is discovered for the first time that the B exciton peak for WSe_2_ quenches with increasing the temperature.

Along with changing of the luminescent intensity, the direct transition in the heterostructures experiences a large redshift with increasing the temperature, as shown in Fig. 4(f–h). Such behavior is similar to the response of conventional semiconductors under high temperature, which result from the increased electron-phonon interactions and slight changes in bonding lengths. Thus, it provides a method to evaluate temperature of the semiconductor^38^,^39^. By employing a modified Varshni relationship, the temperature dependence of the PL peak position is fitted using^40^,^41^


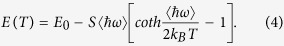


where *E*_0_ is the emission energy at zero absolute temperature, *S* is the Huang-Rhys factor that represents the coupling strength of exciton-phonon, 〈*ħω*〉 is the average phonon energy, *ħ* and *k*_*B*_ are the Plancks and Boltzmann constant, respectively. As shown in Fig. 4(f–h), the fitting parameters are extracted. Table 1 lists the fitting values of *E*_0_, *S*, and 〈*ħω*〉 for MoS_2_, WS_2_, and WSe_2_ in the heterostructures. By comparing these parameters, especially *S*, between the TMD in the heterostructures and monolayer^38^,^42^, the contribution of the interlayer coupling can be informed. Moreover, with the help of this expression, it is possible to derive the temperature difference of the above heterostructure in the device where the PL is measured by comparing the emission energy difference^38^,^43^. However, from Fig. 4(g,h), the peak P_*indiret*_ do not show an obvious redshift as the P_*M*_, 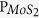, or 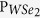, which is due to the gradual decreasing VBM at the Γ point caused by increasing the interlamellar spacing with the temperature. As shown in Figure S1, this decrease of VBN at the Γ point results in a blueshift of the indirect transition in double layer WS_2_.

## Conclusions

To summarize, the interlayer coupling tuning of 2D heterostructures formed with CVD-grown monolayer MoS_2_, WS_2_, and WSe_2_ is carried out by thermal annealing process and variable-temperature experiment. By comparing the tuning methods, the conclusion is made that the temperature is an extremely sensitive factor to the interlayer coupling. Based on the DFT calculations, an interesting phenomenon is found that MoS_2_-WS_2_, MoS_2_-WSe_2_, and WS_2_-WSe_2_ heterostructures turn into direct-gap semiconductors from indirect-gap semiconductors with increasing the interlayer space. Furthermore in the MoS_2_-WS_2_, MoS_2_-WSe_2_, and WS_2_-WSe_2_ heterostructures, the electronic structure changing process with interlayer spacing is different from each other. Our results highlight the significance of interlayer coupling in tuning the light emission of TMDs and offer a general route to prepare large-area TMD tandem structures for fundamental study as well as electronic and photovoltaic applications.

## Methods

### Synthesis of MoS_2_, WS_2_ and WSe_2_

The MoS_2_ monolayers were grown by low-pressure CVD technique for 10 min. The SiO_2_/Si substrates were cleaned using piranha solution (a volumetric mixture of 3:1 of 98% H_2_SO_4_ to 35% H_2_O_2_), then placed in the center of the quartz tube′s heating zone and heated to 850 °C in argon atmosphere to restrict further oxidation. The molybdenum trioxide (MoO_3_) powder was loaded in front of the substrates, while the sulfur powder was placed in the front of the quartz tube, which was heated to 200 °C by a heating band as shown in Figure S3. The WS_2_ and WSe_2_ monolayers were separately fabricated by constant-pressure vapor phase deposition method at 1100 °C and 1200 °C in argon atmosphere for 10 min.

### Transfer Method

The TMDs obtained by mechanical exfoliation have a small flake size, and it is not possible to create large scale heterostructured materials. Thus, the heterostructures here were prepared from chemical vapor deposition (CVD) growth (see Supporting Information Figure S3).

Figure S4 illustrates the polystyrene (PS) film transfer technique schematically. The process started by coating a layer of polymer on top of the as-grown TMDs. The polymer acted as carrier layer, which can help with the handling of the atomically thin materials. The 10 wt% PS was dissolved in toluene. It was spin-coated on the CVD grown monolayer TMDs/SiO_2_/Si with a speed of 3500 rmp for 1 minute. Then, the samples with coating layer were baked at 90 °C for 1 hour. This was designed to evaporate the toluene and eliminate air bubbles formed at the interface of monolayer TMDs and PS, so that the adhesion is increased. This was followed by a gentle poking made by a glass cutter at the edge of the PS/TMDs/SiO_2_/Si to expose SiO_2_, by this way, the water penetration in the next step can be improved. Then, the PS/TMDs/SiO_2_/Si was sank into a large drop of deionized water in the hydrophobic plastic petri dishes gently. This water penetration operation was repeated for several times, until the PS/TMDs film was floated on the top of the water because of the repulsion between the PS and water. Next, the substrate was taken away, and another TMDs/SiO_2_/Si was put into the water from the side. After that, the system was dried by simply sucking the water droplet away with a paper towel. During this process, the PS/TMDs was fixed above the TMDs/SiO_2_/Si with a tweezers. After the PS/TMDs was transferred to the TMDs/SiO_2_/Si, the bubbles in the transferred assembly were purged out with the hydrogen. In order to combine the heterostructures better, the samples were baked at 90 °C for 30 min to remove water residues, and then at 120 °C for 30 min to spread the polymer for the elimination of possible wrinkles. Finally, the transferred assembly was soaked in toluene for at least 2 hours with the toluene changed several times during this time. It was worth noting that the obtained heterostructures were much cleaner than the conventional polymethyl methacrylate (PMMA) transfer techniques with reduced transfer film residue and improved quality (Figure S5)^44^,^45^. Besides, the new transfer technique has the advantage of high efficiency on account of the regardless of the thickness of spin-coating layer.

### Raman/PL Spectroscopy

Temperature dependent PL experiments were carried out by a Jobin-Yvon LabRAM HR 800 micro-Raman spectrometer and a THMSE 600 heating/cooling stage (Linkam Scientific Instruments) in the temperature range from −196 °C to 2 °C with a resolution of 0.1 °C. The heterostructures was excited by the 488 nm line of an Ar laser with the output power of 20 mW and recorded in back-scattering geometry with a resolution of better than 1 cm^−1^. The laser beam was focused through a 50× microscope with a working distance of 18 mm. An air-cooled CCD (−70 °C) with a 1024 × 256 pixels front illuminated chip was used to collect the scattered signal dispersed on 1800 grooves/mm grating for Raman and 600 grooves/mm grating for PL^46^. Peaks of the bare spectra are assigned by using the supporting software NGSLabSpec designed by JobinYvon.

### Computational Details

Our density functional theory (DFT) calculations were performed with plane-wave pseudopotentials from the calculate method of quantum mechanics^47^,^48^. The generalized gradient approximation (GGA) for exchange-correlation term was employed, and the functional was adopted for structure optimization and band gaps calculation^49^. The selected pseudopotential is Ultrasoft Pseudopotentials. To acquire accurate results for bilayer structures, DFT-D approach was included with the Ortmann-Bechstedt-Schmidt (OBS) vdW correction^50^, which gives the structural parameters in good agreement with experimental values. A planewave cutoff energy of 320 eV and a 5 × 5 × 1 grid of Monkhorst-Pack points were employed to ensure good convergence of the computed structures and energies. Geometry optimization was determined using the Broyden-Fletcher-Goldfarb-Shenno (BFGS) minimization technique, with thresholds of converged structure of energy change per atom smaller than 10^−5^ eV/atom, and displacement of atoms during geometry optimization no more than 0.001 A. The tolerance in the self-consistent field (SCF) calculation was 1.0 × 10^−6^ eV/atom. All calculations were carried out using a 1 × 1 supercell with vacuum thickness not smaller than 17 Å and spin-orbit coupling was not included.

## Additional Information

**How to cite this article:** Wang, F. *et al*. Tuning Coupling Behavior of Stacked Heterostructures Based on MoS_2_, WS_2_, and WSe_2_. *Sci. Rep.* **7**, 44712; doi: 10.1038/srep44712 (2017).

**Publisher's note:** Springer Nature remains neutral with regard to jurisdictional claims in published maps and institutional affiliations.

## Supplementary Material

Supplementary Information

## Figures and Tables

**Table 1:** Fitting parameters of the PL peak energy as a function of temperature extracted in Fig. 4(f–h).

Samples	excition	*E*_0_ (eV)	*S*	〈*ħω*〉 (meV)
MoS_2_-WS_2_	P_*M*_	1.92	2.78	9.60
MoS_2_-WSe_2_	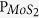	1.93	1.91	8.63
	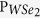	1.71	2.23	6.42
WS_2_-WSe_2_	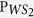	2.00	3.72	7.84
	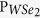	1.72	2.99	6.11
