# Supplementary material for: Tuning Coupling Behavior of Stacked Heterostructures Based on MoS2, WS2, and WSe2
Source: Sci Rep. 2017 Mar 17;7:44712. doi: 10.1038/srep44712 (PMC5356184; doi:10.1038/srep44712)
Supplement: Supplementary Information [file srep44712-s1.pdf]

Supporting Information

Tuning Coupling Behavior of Stacked Heterostructures Based on MoS_2, WS_2, and WSe_2

Fang Wang, Junyong Wang, Shuang Guo, Jinzhong Zhang, Zhigao Hu,* and Junhao Chu

Technical Center for Multifunctional Magneto-Optical Spectroscopy (ECNU), Shanghai

Department of Electronic Engineering, East China Normal University, Shanghai 200241, China

*Author to whom correspondence should be addressed. Tel.: +86-21-54345150. Fax: +86-21-54345119.

Electronic mail: zghu@ee.ecnu.edu.cn

(Dated: January 24, 2017)

Temperature dependent PL spectral of the double layer WS_2

Double layer WS_2 is an indirect bandgap semiconductor. Figure S1 (a) shows that the PL spectral of double layer WS_2. As shown in Figure S1 (b) and (c), the PL intensity of the direct transition decreases with increasing the temperature, and the PL peaks experience a large redshift. However, the PL peaks of the indirect transition experience a blueshift with increasing the temperature. Figure S1 (d) shows the color contour map corresponding to the typical PL behavior of WS_2 between 1.53 eV to 2.1 eV. The redshift and intensity attenuation are obvious.

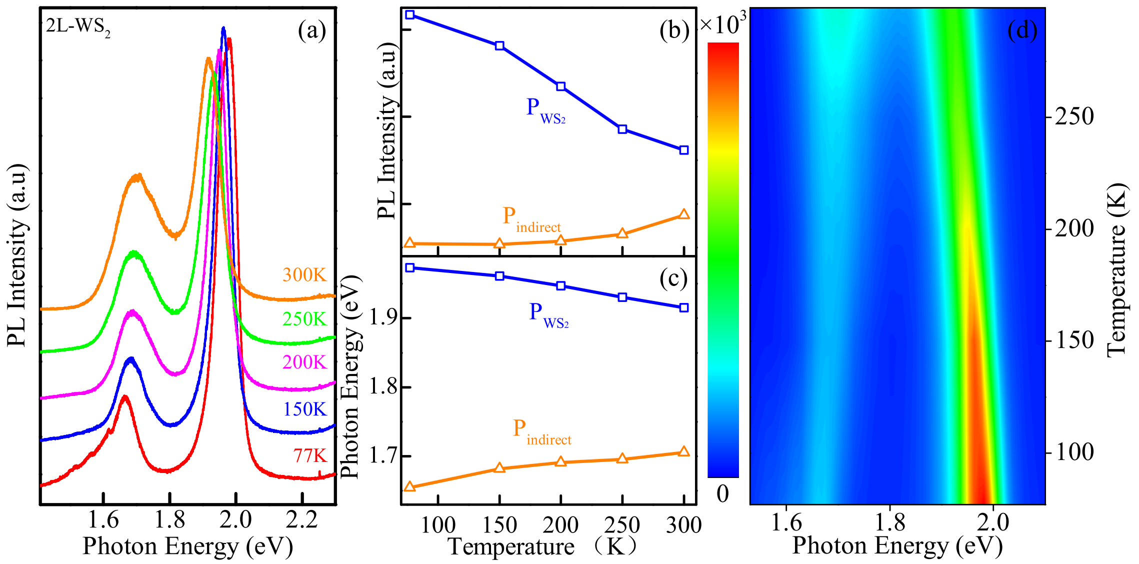

Figure S 1: (a) Temperature dependent PL of double layer WS_2. (b) Fitted PL intensity and (c) peak position of the direct and indirect transition of WS_2. (d) Color contour map of PL spectra as a function of temperature between 1.53 eV and 2.1 eV, The colour scale right corresponding to the intensity of the PL spectra.

Temperature dependent PL spectral of the monolayer MoS_2

Figure S2 (a) shows that the PL spectral of monolayer MoS_2. As shown in Figure S2 (b) and (c), the PL intensity decreases with increasing the temperature, and the PL peaks experience a large red shift. Figure S2 (d) shows the color contour map corresponding to the typical PL behavior of MoS_2 between 1.6 eV to 2.2 eV. The redshift and intensity attenuation are obvious.

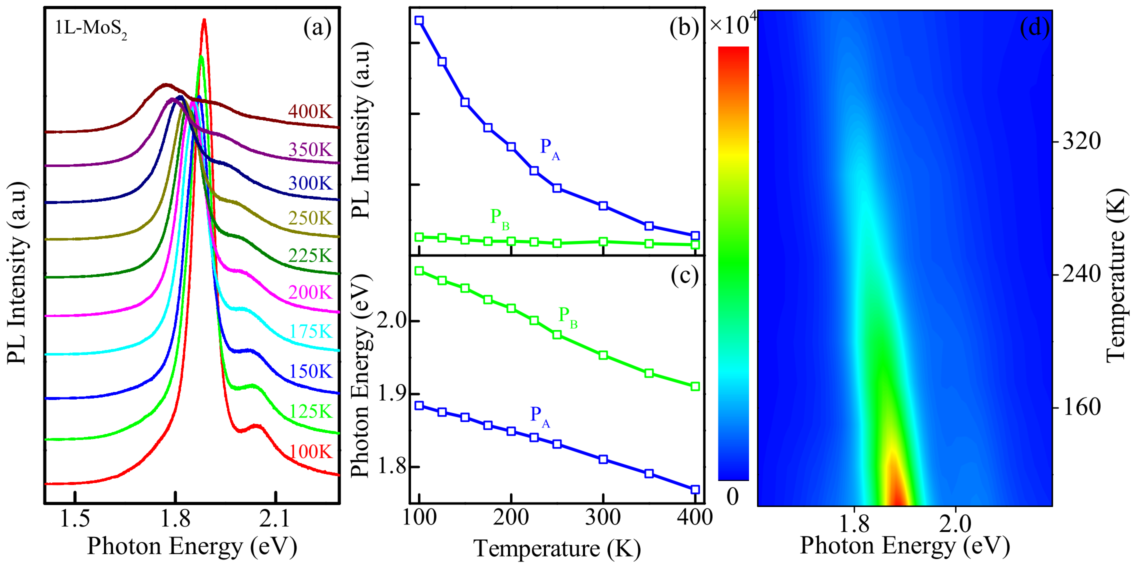

Figure S 2: (a) Temperature dependent PL of monolayer MoS_2. (b) Fitted PL intensity and (c) peak position of the MoS_2 A and B excitons versus temperature. (d) Color contour map of PL spectra as a function of temperature between 1.6 eV and 2.2 eV, The colour scale right corresponding to the intensity of the PL spectra.

CVD growth technique

The MoS_2 monolayers were grown by low-pressure CVD technique. The molybdenum trioxide (MoO_3) powder was loaded in front of the SiO_2/Si substrates in the quartz tube and heated to 850 °C, while the sulfur powder was placed in the front of the quartz tube which was heated to 200 °C by a heating band as shown in Figure S3. The WS_2 and WSe_2 monolayers were fabricated by constant-pressure vapor phase deposition method at 1100 °C and 1200 °C in argon atmosphere. The growth time is 10 min.

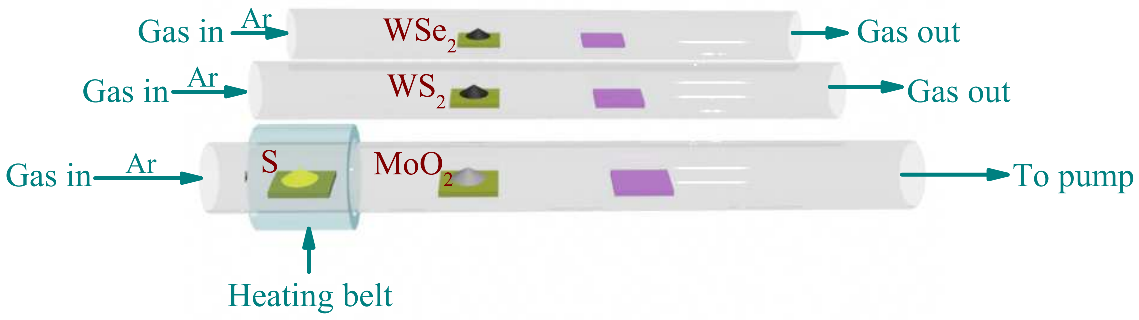

Figure S 3: Schematic illustration of the CVD system for MoS_2, WS_2, and WSe_2 growth.

Transfer processes

In a typical transfer process, 10 wt % PS dissolved in toluene was spin-coated (3500 rpm for 1 minute) on as-grown TMDs films on the SiO_2/Si [Figure S4(b)]. This was followed by a baking at 90 °C for 1 hour to facilitate intimate adhesion of the PS layer with the TMDs film. The edge of the PS film was scribed with glass cutter to lead in deionized water. Then, the samples were soaked into and taken out of the deionized water for several times until the polymer layer with TMDs broke away from the substrate [Figure S4(c)]. Next, another TMDs/SiO_2/Si was put under the polymer layer into the water from the side[Figure S4(e)]. After sucking the water droplet away with a paper towel [Figure S4(f)], we purged out the bubble with the hydrogen, and baked the transferred assembly at 90 °C for 30 min and then at 120 °C for 30 min. At last, the PS coating was removed with toluene.

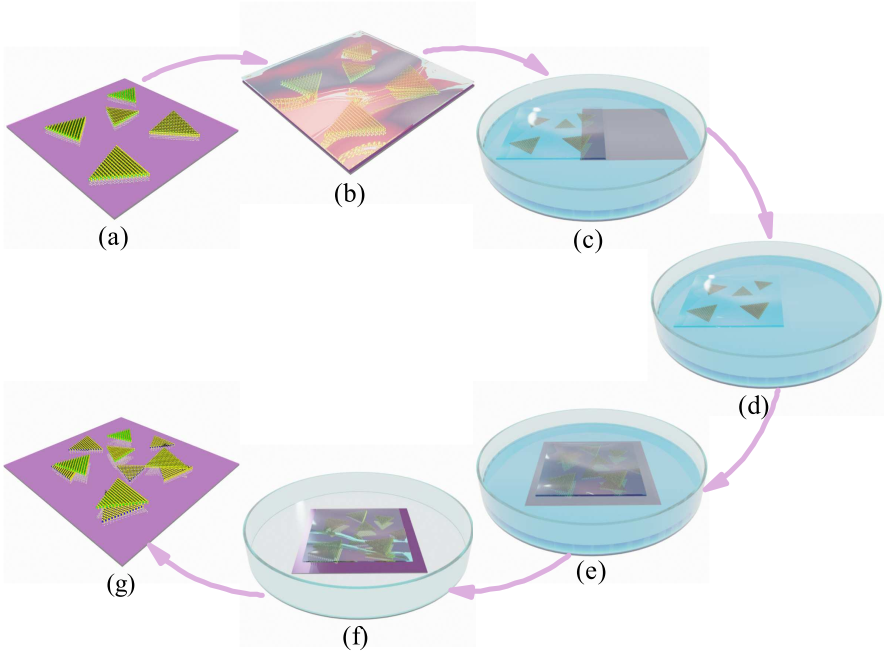

Figure S 4: Schematic illustration of the PS film transfer processes.

Comparison of the PS and PMMA transfer techniques

The comparison is made between the PS and conventional PMMA film transfer techniques. It is obvious that the heterostructures obtained with the PS film transfer technique is much cleaner than the PMMA transfer techniques with reduced transfer film residue and improved quality.

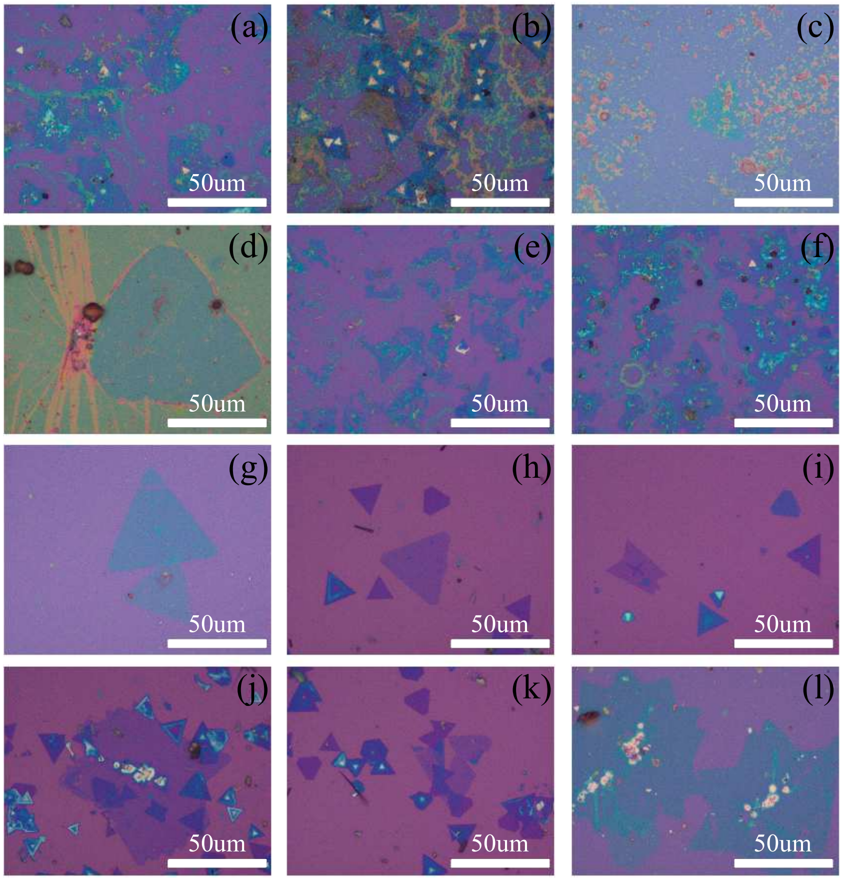

Figure S 5: (a-f) Optical microscope image of PMMA film transferred TMDs heterostructures. (g-l) Optical microscope image of the conventional PS film transferred TMDs heterostructures.
